# Concerning Stiffness of the Spine

**Published:** 1907-04-20

**Authors:** William Bennett


### Hospital Clinics.

### CONCERNING STIFFNESS OF THE SPINE.

By SIR WILLIAM BENNETT, K.C.V.O.

A Post-Graduate Lecture delivered at the London School of Clinical Medicine (Seamen's Hospital). *Concluded from p. 29.*

### Intermittent or Relapsing Stiffness.

*Neuro-Mimesis in Relation to Spinal Rigidity.*—Neuro-mimesis, the nervous mimicry of disease, does not, I fancy, attract so much attention now as it did 20 or 25 years ago, although, judging from personal experience, it is quite as important a clinical factor now as it was then. I may, perhaps, be pardoned if I remind you that the so-called intelligence displayed by young children is largely due to the results of pure imitation, and that during the early years of life the tendency, especially in girls, to imitate the characteristics and peculiarities of those with whom they continually associate is very strong; this imitative faculty being particularly attracted in certain subjects by any characteristic which is especially pronounced. Occasionally it happens that an unusually susceptible child, thrown constantly in contact with another individual, whether child or adult, suffering from some deformity or peculiarity, will unconsciously imitate the peculiar characteristic in a remarkable way. The commonest conditions thus imitated are, in my experience, disease of the hip, and Pott's disease. Confining ourselves to the consideration of the subject of this lecture, I have seen cases of slight angular curvature at the cervico-dorsal junction so accurately imitated as to deceive very shrewd observers. A peculiarity, however, of the spurious condition is that it occurs in precisely the same position, and assumes as nearly as possible the same characteristics, as the original case, whereas in the case of organic disease, especially tubercle occurring in a second member of the same family it is very rarely indeed of precisely the same form and distribution as the first—the very similarity of the two cases should, in fact, arouse suspicion as to the reality of the second. The stammering test will also invariably exclude the existence of organic disease, and if any ultimate doubt remains, the administration of an anæsthetic will finally settle the question by completely removing the rigidity if the case be one of the spurious kind. The moral of this matter is as follows: In a case of rigidity of the spine with, perhaps, apparent slight deformity, the mere fact that another member of the family has had tuberculous disease of the vertebral column is not necessarily any evidence that the second case is of the same nature, especially if the manifestations in the second case are precisely similar to those in the first; in such circumstances the possibility of neuro-mimesis should always be borne in mind.

### An Instructive Case.

Some years ago a girl 14 years old was brought to me for an opinion concerning a curious stiffness about the neck and shoulders, and a peculiarity in gait which she had gradually developed in the course of eight or nine months. The attitude was singularly restrained—the head was thrust a little forward, the chin being slightly raised; on turning from side to side the head moved with the shoulders and the neck seemed quite stiff. The general attitude was precisely that of Pott's disease in the cervico-dorsal region. The girl was well nourished, very intelligent, and in apparently good general health. She complained of no actual pain, but readily became tired, and had given up any attempt to play games. On removing the clothes the position of the head and neck was as already described, the lower cervical and upper dorsal spine was apparently quite stiff, the vertebra prominens projected much more than is usual (which suggested a condition of slight angular curvature) there was no tenderness or thickening of any kind, the bony outlines could be clearly made out, and the ordinary bending test only confirmed the impression of stiffness of the column. The stammering test I did not use, as I had not then become acquainted with it. I felt, however, that there was something deceptive about the case, and therefore examined the child under an anæsthetic, with the result that all the symptoms disappeared, the spine becoming during the anæsthesia entirely natural, whilst upon recovery from the anæsthesia the abnormal condition was resumed. What the condition during ordinary sleep was I do not know, as so far as I can recollect I never had an opportunity of satisfying myself on that point. The case puzzled me a good deal at the time; there was clearly no organic disease, and as a hysterical manifestation it was most unusual. It transpired on further inquiry that the girl had for a year or more before I saw her been constantly in the company of a patient who had tuberculous disease of the cervico-dorsal spine, and who wore a leather support having a collar round the neck to support the head and chin. This appeared to settle all doubt as to the nature of the case, which was one of nervous mimicry of the most striking kind. The position in which the patient held her head was, it will be noted, precisely that in which the head of a patient would be whilst wearing a spinal support of the kind mentioned. Removal from the associations under which the abnormality developed, and the use of massage and rational exercises, were followed by great, although somewhat slow, improvement.

The recognition of these cases is of considerable importance, for unless proper means are taken to correct the perverted function the abnormality may become permanent, a result which is all the more likely if in such a case a diagnosis of organic disease be made and the usual treatment by rest and fixation employed. There is no limit to the eccentricities which are shown by some of these patients. I remember a remarkable case at St. George's Hospital, in which a clearly neuro-mimetic talipes equino-varus in a girl disappeared under an anæsthetic, and was found on the recovery from the anæsthetic to have been transferred to the opposite foot; the mistake, however, was not long in being corrected, for in a few hours the abnormality was again located in the foot originally concerned.

### Reflex Irritation as a Cause of Intermittent Spinal Rigidity.

The reflex disturbances resulting from intestinal worms in children are so manifold as to be almost unlimited; at the same time I do not know that occasional spinal rigidity is generally recognised as one of them. There is no doubt, however, that intestinal worms do produce this complication, and sometimes in a very alarming degree. A case, for example, came under my notice in which a relapsing spinal rigidity of a very pronounced kind disappeared entirely after the passage of a large mass of tape-worm.

*Movable Kidney.*—The following case speaks for itself. A young woman, 28 years old, of a spare and apparently healthy habit, suffered almost continuously from pain across the lower part of the spine; the pain, as a rule, was dull, but sometimes it became acute; stooping was so painful that she found it as a rule almost impossible to pick up any object from the ground. Upon examination the whole spine below the middle of the dorsal region was stiff when she bent; there was neither deformity nor tenderness, but the erectores spinæ were very hard. The stammering test could not be applied, as the pain on stooping was too great to allow the necessary attitude to be maintained for a sufficiently long time. On examining the abdomen the recti were rather rigid, and at first resented handling. A little gentle manipulative massage, however, led to their relaxation, and on the right side a distinct mass could be felt, which suddenly slipped away in the characteristic manner of a movable kidney. This was followed by a sense of relief like that which was usually felt when sudden or rapid modification in the symptoms happened. It therefore seemed pretty clear that the whole trouble might be due to a movable kidney which was prevented from returning to its normal site by some band or constriction after it had become displaced. At all events the probability of this view being correct was strong enough to justify the recommendation of an operation to fix the kidney. This was accordingly done, with the result that no further spinal symptoms had occurred up to the time when she was last seen—three years after the operation.

### Calculi as Causative Factors.

*Renal Calculus.*—Spinal rigidity, generally of the relapsing type, is met with in cases of renal calculus. I was consulted by a medical student, 24 years old, on account of a persistent pain in the spine over the dorso-lumbar region, which varied in degree from a dull ache to an acute agony; it was increased by movement, especially jolting. There was no tenderness or deformity, and violent succussion of the shoulders caused no increase of discomfort; on the right side the pain passed round the belly " like half a girdle." When I first saw him the lower half of the vertebral column was quite stiff in all movements, and he walked in the constrained manner of a man with a stiff spine. The stammering test led to a quivering in the erector spinæ, but the pain was so much increased by stooping that the test could not be fully carried out; but what I felt, was sufficient to assure me that no organic disease of the spine existed. There were no symptoms referred to the kidney, and the urine was normal saving the existence of a few crystals of oxalate of lime. A week later, although the symptoms of pain, etc., were much the same as before, the spine was was quite supple and the constrained gait in walking, which was present before, no longer existed. The renal region now was a little tender, and the muscles resented pressure to a slight extent. In five successive examinations the spine was quite stiff in two, quite supple in three. In the renal tenderness no change occurred, and the urine remained normal. An *x*-ray exposure was made, and there seemed to be an opacity in the right kidney, but the case occurred in the earlier *x*-ray period, and the result was too indefinite to be conclusive. I finally explored the right kidney and removed an oxalate of lime stone as large as a hazel nut. The operation proved entirely successful, as all the symptoms disappeared and did not recur. The patient had unfortunately, in attempting to relieve the pain, contracted the morphia habit before I saw him, and the ultimate result was lamentable.

*Stone in the Ureter.*—Within the last eighteen months I have seen a remarkable case of this kind in which spinal rigidity and pain were the conditions complained of. The patient was a stoutish, middle-aged man, of an obviously gouty habit. The pain, which was constant, was referred to the lumbar region, and was at times intense. When first seen, rigidity of the lumbar spine was apparently complete, and the lower dorsal vertebræ moved less freely than was normal. The stammering test established the fact that there was no organic disease of the spine. On one occasion the rigidity was altogether absent. The *x*-rays showed a stone of small size in the ureter. This, under the flushing-out treatment, was subsequently passed, and the symptoms complained of did not occur again.

### Temporary or Transient Rigidity.

It is manifest that transient rigidity may arise from several conditions affecting the vertebral column apart from those the result of reflex causes. The only cause, however, which I need mention here is rickets. The general belief that the rickety spine is a supple spine is universally taught, and rightly so; but it is nevertheless a fact that during the gastro-intestinal disturbance in rickets the spine may for a period be rigid.

In conclusion it may not be amiss to remind you that whilst on the one hand spinal rigidity is not necessarily an indication of disease of the vertebral column, its absence, on the other hand, does not show that the column is free from lesion, since in some cases of tubercle considerable changes in the condition of the column may occur without its suppleness being interfered with.

